# Community-Level Awareness of Proper Immediate Steps Regarding Ocular Chemical Injury in Qassim Region, Saudi Arabia

**DOI:** 10.7759/cureus.47023

**Published:** 2023-10-14

**Authors:** Shahad A Alotaibi, Atheer S Alnayf, Enas B Taha, Bushra Mohandes, Abdulaziz M Alhadlaq, Eman A Alotaibi

**Affiliations:** 1 College of Medicine, Sulaiman Al Rajhi University, Al Bukayriah, SAU; 2 College of Medicine, Sulaiman Alrajhi University, Bukayriah, SAU; 3 Department of Ophthalmology, College of Medicine, Qassim University, Qassim, SAU; 4 Department of Family and Community Medicine, Unaizah College of Medicine and Medical Sciences, Qassim University, Unaizah, SAU

**Keywords:** general public awareness, eye emergency, community awareness, chemical eye injuries, eye injuries

## Abstract

Background

An ocular chemical injury is a critical medical condition that results in harm to many components within the orbit, including the epithelium, cornea, and anterior segment. Most accidents tend to transpire among individuals in younger age groups, primarily as a result of their exposure to hazardous conditions either within their domestic environment or in occupational settings.

Objective

The objective of this study is to evaluate the extent of community awareness of chemical eye injuries and the appropriate measures for urgent care, focusing on different age groups within the Al-Qassim region.

Methods

A cross-sectional study was utilized in this research, employing data from a sample of 384 individuals residing in Qassim. The participants completed a self-administered questionnaire, which was administered online and ensured anonymity. The questionnaire used in this study had been previously validated.

Results

The study included a total of 384 participants who were not affiliated with the medical field, selected from the Al-Qassim region. Most of the respondents demonstrated fairly good level of awareness regarding ocular chemical injury (69%). There was a statistically significant relationship between age and the level of awareness (p-value=0.00001<0.05). Most participants believed that ocular chemical injury could cause ocular complications (93.8%) and identified chloride and detergents (92.2%) as the main materials that cause ocular injuries.

Conclusion

Overall summary, this study’s findings suggest that there exists a moderate degree of knowledge among the public regarding chemical injuries to the eye and the appropriate rapid response to such injuries. Majority of the community members were aware that ocular chemical injury can cause ocular complications, blindness, perforation, scarring, and cataracts. Additionally, chloride and detergents were found to be the most common causes of ocular injuries among the population. The study found the most common immediate action when exposed to ocular chemical injury was to irrigate the eye with large amount of water. The study established a statistically significant association between the age and awareness about ocular chemical injury; thus, age appears to be a key factor influencing the amount of awareness seen.

## Introduction

An ocular chemical injury is a critical medical condition that results in harm to many components within the orbit, including the epithelium, cornea, and anterior segment. The majority of accidents tend to transpire among individuals in younger age groups, primarily as a result of their exposure to hazardous conditions either within their domestic environment or in occupational settings [1]. The severity of the injury is such that it has the potential to result in visual impairment. Nevertheless, by implementing appropriate management protocols, the likelihood of blindness can be substantially mitigated [2].

Chemical injuries account for approximately 7% of work-related eye injuries that receive medical treatment in hospital emergency departments in the United States [3]. Given the potential lack of knowledge among the Saudi population on the appropriate measures and procedures to be followed in the event of a chemical injury to the eyes, it is important to emphasize that ocular chemical injuries represent genuine eye emergencies that necessitate prompt assessment and intervention. Ocular or thermal burns account for approximately 7.7% to 18% of all reported ocular trauma incidents [4]. Chemical injuries encompass both alkaline and acidic damage. The prevalence of alkaline burns is higher as a result of the extensive use of alkaline compounds in both industrial and domestic cleaning agents [5]. Consequently, these burns often lead to more substantial and serious injuries [5]. Immediate initiation of therapy for chemical burns is crucial and should be promptly administered at the site and time of the injury. The recommended course of action is to thoroughly irrigate the afflicted eye(s) with a non-caustic fluid, if one is readily accessible at the site of damage, and to continue this irrigation process during transportation to the medical facility [6]. It is recommended that irrigation be maintained at the medical facility until the pH of the ocular surface has returned to a normal range of 7.0 to 7.2 [7].

Chemical burns affecting the eye account for around 11.5% to 22.1% of ocular injuries [8]. Numerous investigations have evaluated the comprehensive prevalence and causation of ocular chemical damage. According to a meta-analysis conducted in 2021, the reported incidence rate of the condition ranged from 5.1 to 50 per 100,000 individuals’ year across multiple nations [9]. There exists a concept positing that despite the potential for vision impairment resulting from such injuries, there is a notable dearth of awareness within the community [10]. Numerous researches have been conducted to evaluate the level of knowledge on the reversible management of chemical eye damage [10,11]. According to a prior investigation, it was seen that females exhibited a notably greater level of knowledge compared to males [11]. Additionally, individuals with higher educational attainment displayed a higher degree of knowledge in contrast to those with lower educational levels [12].

According to a study conducted on the Saudi community, it was shown that a significant proportion, specifically 20.3% of the population, employs improper approaches when it comes to handling burns [13]. This study will be unique in its focus on the Saudi population, its exploration of disparities in knowledge, and its investigation into the awareness and local management practices regarding ocular chemical injuries. By addressing these specific aspects, the study aims to contribute valuable insights into this critical healthcare issue within the Saudi context and provide a foundation for targeted interventions and policies to improve public awareness and emergency response.

## Materials and methods

Study area/setting

A cross-sectional study involving non-medical students was conducted between the period of May 2023 and August 2023 within the geographical borders of the Al-Qassim region.

Study subjects

The study subjects were the general non-medical population and the study aimed to determine the extent to which individuals adhere to recommended reversible treatment measures aimed at preventing blindness in cases of such injuries.

Sample size

The Raosoft sample size calculator (http://www.raosoft.com/samplesize.html) was used to calculate the sample size based on the total population and estimated population in the Al-Qassim region, which is approximately 10,50,000, according to the General Authority for Statistics in the Kingdom of Saudi Arabia. The desired level of confidence was set at 95%, with a margin of error of 5% and a response distribution of 50%. Consequently, the minimum required sample size was established as 385.

Sampling technique

We adopted random sampling technique to ensure the general non-medical population had an equal chance of selection.

Data collection methods, instruments used, and measurements

Upon receiving ethical approval, an online questionnaire was disseminated within the selected population through social media platforms. Participation in the survey was entirely voluntary. The questionnaire underwent adaptations and expansions to ensure alignment with the specific objectives of the study. It encompassed various sections, including a social demographic segment, to gather comprehensive data for the research. The face validity of the instrument was evaluated by two occupational health specialists who reviewed the questionnaire. Reliability based on Cronbach’s alpha produced an acceptable value for the final version of the questionnaire. The distribution of this self-administered questionnaire was conducted through internet platforms, specifically utilizing social media websites. The study population consisted of individuals residing in the Al-Qassim district who were between the ages of 13 and 65 and did not have a medical background. This inclusivity allows for a comprehensive examination of knowledge and practices related to ocular chemical injuries across different age groups. Understanding how various age cohorts perceive and respond to such incidents is valuable for tailoring educational initiatives.

Data management and analysis plan

Following the collection of data, a manual process was undertaken to validate and code the information within an Excel spreadsheet. The data were coded before entry into the software program. Descriptive statistics were applied, summarizing the data in terms of frequency and percentage. Chi-square tests were used for analyzing categorical variables and to determine the association between the groups, with significance set at a P-value of 0.05.

Ethical considerations: informed consent

The authors have no conflict of interest, and the work was not supported or funded by any drug company. Ethical approval was sought from the Research Ethics Committee at Qassim Health (No. H-04-002). Written consent was obtained from each participant before the study was conducted. All collected data were kept confidential and used solely for research purposes.

## Results

According to Table 1, 384 non-medical individuals were included in this study from the Al-Qassim region. The commonest age group was found to be 21-30 years 173 (45.1%), followed by 13-20 years 68 (17.7%), while the least common age group was found to be 61-65 years 13 (3.4%), followed by 41-50 years 37 (9.6%).

**Table 1:** The age distribution of respondents (N=384). Age of respondents presented in frequencies (n) and proportion (%)

Variables	Category	Frequency and proportion n (%)
Age (years)	13-20	68 (17.7%)
	21-30	173 (45.1%)
	31-40	53 (13.8%)
	41-50	37 (9.6%)
	51-60	40 (10.4%)
	61-65	13 (3.4%)

In Table 2, regarding whether ocular chemical injury can cause ocular complications, the majority of respondents agreed 360 (93.8%). When asked what damage an ocular chemical injury can cause, the commonest answer was blindness 302 (78.6%), followed by perforation 182 (47.4%), scarring 139 (36.2%), and cataracts 133 (34.66%). Regarding the materials that commonly cause ocular injuries, the commonest answer was chloride and detergents 354 (92.22%), followed by battery materials 277 (72.1%), then vinegar 174 (45.3%), and lastly, water 10 (2.66%). When the respondents were asked about the immediate action when exposed to ocular chemical injury, the most common answer was irrigation of the eye with a large amount of water 222 (57.8%), followed by going to the emergency department 130 (33.9%), and then irrigation of the eye with a small amount of water 17 (4.4%). Regarding the substances that respondents might use in case of ocular chemical injury, 263 (68.5%) said they would use water if the substance was alkalic or acidic, while 106 (27.6%) did not know the answer. The majority of respondents 277 (72.1%) agreed that management varies according to the substance of the ocular injury, while 27 (7%) disagreed. When asked about the optimal duration of eye washing following ocular chemical injury, 203 (52.9%) said < 5 minutes, followed by 5-15 minutes 171 (44.5%), while 10 (2.6%) said 30 minutes or more. 121 (31.5%) of respondents stated that the best way to wash the eye is by passing water from the middle part of the face to the tip of the eye, while 90 (23.4%) said by passing water from the tip of the eye to the middle part of the face, and 67 (17.4%) said by putting water in a cup and covering the eye with it. The majority of respondents 253 (65.9%) agreed that the severity of pain indicates the severity of the ocular chemical injury, while 57 (14.8%) disagreed. The majority 338 (88%) believe that eye rubbing after chemical exposure increases the severity of the ocular injury, while 7 (1.8%) disagree. When asked about the most serious sign of ocular injury, the most common answer was eyelid sticking 131 (34.1%), followed by eye white discoloration 110 (28.6%), and then severe pain 108 (28.1%). The majority of respondents 297 (77.3%) do not believe that wearing contact lenses protects against ocular chemical injury, while 25 (6.5%) do. Regarding whether the contact lens should be removed in case of ocular chemical injury, 274 (71.4%) said yes, 26 (6.7%) said no, and 84 (21.9%) did not know. 340 (88.5%) of respondents believe that wearing goggles lowers the risk of ocular injury, while 23 (6%) do not. When asked if respondents should wash their hands before touching the eye after handling chemical materials, 365 (95.1%) agreed, while 7 (1.8%) disagreed.

**Table 2:** Awareness of respondents about ocular chemical injury. Respondents’ awareness about ocular chemical injury presented in frequency (n) and proportions (%)

Statement/Question	Variable	Frequency and proportion n (%)
Ocular chemical injury can cause ocular complications	I agree	360 (93.8%)
	I don’t agree	6 (1.5%)
	I don’t know	18 (4.7%)
What damage can ocular chemical injury cause?	Blindness	302 (78.6%)
	Perforation	182 (47.4%)
	Scarring	139 (36.2%)
	Cancer	115 (29.9%)
	Keratoconus	105 (27.3%)
	Cataract	133 (34.6%)
What are materials that commonly cause ocular injuries?	Chloride and detergents	354 (92.2%)
	Battery materials	277 (72.1%)
	Vinegar	174 (45.3%)
	Water	10 (2.6%)
What is your immediate action when exposed to ocular chemical injury?	Irrigation of the eye with a large amount of water	222 (57.8%)
	Going to the emergency department	130 (33.9%)
	Irrigation of the eye with a small amount of water	17 (4.4%)
	Using eye drops from a pharmacy	7 (1.8%)
	Covering the eye	8 (2.1%)
What will you use to treat an ocular chemical injury?	An acidic substance if the injury substance is alkalic	6 (1.6%)
	An alkalic substance if the injury substance is acidic	9 (2.3%)
	Water in both cases	263 (68.5%)
	I don’t know	106 (27.6%)
Management varies according to the substance of ocular injury	I agree	277 (72.1%)
	I don’t agree	27 (7.0%)
	I don’t know	80 (20.8%)
What is the optimal duration of eye washing following ocular chemical injury?	5-15 min	171(44.5%)
	< 5 min	203 (52.9%)
	30 min or more	10 (2.6%)
The best way to wash the eye	Passing water from the middle part of the face to the tip of the eye	121 (31.5%)
	Passing water from the tip of the eye to the middle part of the face	90 (23.4%)
	Put water in a cup and cover the eye with it	67 (17.4%)
	I don’t know	106 (27.6%)
The severity of pain indicates the severity of the ocular chemical injury	I agree	253 (65.9%)
	I don’t agree	57 (14.8%)
	I don’t know	73 (19.3%)
Eye rubbing after chemical exposure increases the severity of ocular injury	I agree	338 (88.0%)
	I don’t agree	7 (1.8%)
	I don’t know	39 (10.1%)
What is the most serious sign of ocular injury?	Eye redness	35 (9.1%)
	Eye white discoloration	110 (28.6%)
	Eyelid sticking	131 (34.1%)
	Severe pain	108 (28.1%)
Do you think that wearing contact lenses protect against ocular chemical injury?	I agree	25 (6.5%)
	I don’t agree	297 (77.3%)
	I don’t know	62 (16.1%)
In case of ocular chemical injury, you should remove the contact lens.	I agree	274 (71.4%)
	I don’t agree	26 (6.7%)
	I don’t know	84 (21.9%)
Do you think that wearing goggles lowers the risk of ocular injury?	I agree	340 (88.5%)
	I don’t agree	23 (6.0%)
	I don’t know	21 (5.5%)
After handling chemical materials, you should wash your hands before touching the eye.	I agree	365 (95.1%)
	I don’t agree	7 (1.8%)
	I don’t know	12 (3.1%)

**Figure 1:**
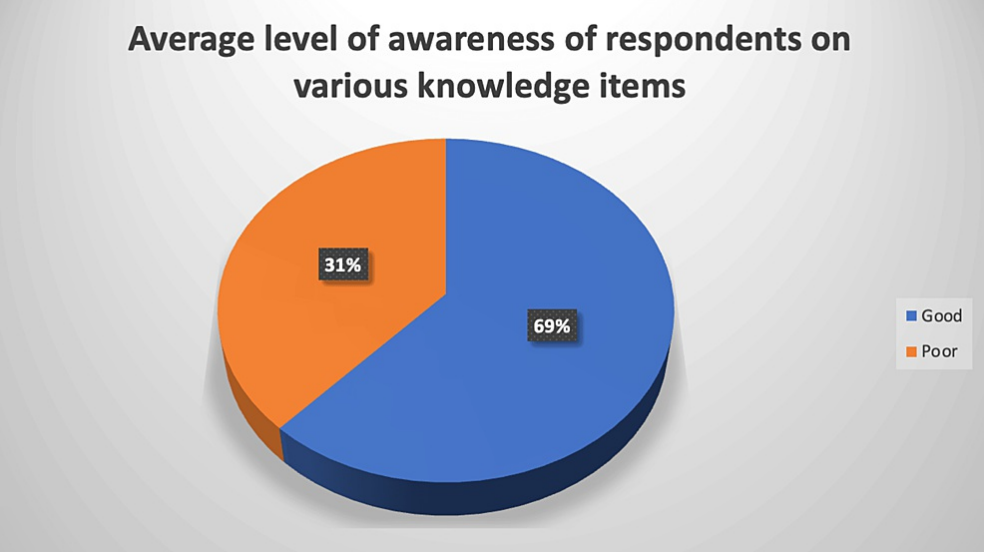
Pie chart depicting the average level of awareness of respondents on various knowledge items. Proportions of respondents’ average level of awareness presented in percentages (%)

The pie chart in the Figure 1 shows there was a moderately good level of awareness among the respondents (69%). Percentile ranks were used to rank the level of awareness considering the 50th percentile (median), we categorized 69% as fairly good.

Table 3 depicts the relationship between social demographic variables and awareness. A statistically significant association was found between age and awareness (P-value < 0.00001).

**Table 3:** The association between social demographic variables and levels of awareness. Statistically significant association between age and awareness established at P< 0.00001 P-value is significant at < 0.05 level

		Awareness		
Variables	Category	Good	Bad	P-value
Age (years)	13-20	64.1%	35.9%	< 0.00001*
	21-30	47.1%	52.9%	
	31-40	46.2%	53.8%	
	41-50	45.0%	55.0%	
	51-60	44.1%	55.9%	
	61-65	1.4%	98.6%	

## Discussion

This study was conducted to measure the non-medical community awareness of different age groups in the Al-Qassim region about chemical injuries to the eye and the immediate management of the injury. The current study reveals a moderately good level of awareness among the respondents. Also, a statistically significant association was found between both age and awareness (P-value < 0.00001). This can be attributed to a variety of factors, including increased life experience, exposure to information over time, and changes in personal priorities. For example, older individuals may become more aware of health-related issues as they age and encounter health challenges [14].

A similar study by Bamahfouz et al. indicated a significant gap in awareness about ocular chemical injuries when compared to participants’ overall knowledge levels. Specifically, most of the respondents (56.62%) demonstrated a good level of knowledge but an insufficient level of awareness regarding previous ocular chemical injury incidents. Notably, the study identified significant associations between higher awareness levels and female participants, individuals aged 20 or older, and Saudi nationality [15]. The difference could be due to diversity in the social demographic variables.

When comparing the results of the current study with the previously done study by Alhothali et al. in Saudi Arabia, the previous study included 1279 responses, and the results revealed that good knowledge was significantly higher among females than males, respondents without a history of eye injury than their peers with injuries, and respondents with higher educational levels than those with lower educational levels. Also, good knowledge was shown by the female gender. In the current study, a statistically significant association was found between age and awareness (P-value < 0.00001) [16]. However, it's important to clarify that these findings do not inherently imply that being female directly causes or affects knowledge. Instead, they suggest a statistical association or correlation between being female and having good knowledge in the context of the study.

Alqassim et al. also conducted research in Saudi Arabia, with most participants between the ages of 18 and 30 (66%) and most participants being female (274) (51.1%) [13]. With an average score of 7.70 out of a maximum of 16, participants demonstrated a general lack of knowledge regarding chemical injuries to the eye. The vast majority of respondents (95.1%) agreed that chemical injury to the eye could cause eye disorders. 317 respondents (59.1%) said they would rinse their eyes with a lot of water if they experienced an ocular chemical injury; 155 (28.9%) said they would go to the emergency room; 40 (7.5%) indicated they would rinse their eyes with a little water; 13 (2.4%) said they would use eye drops; and 11 (2.1%) said they would cover their eyes. On the other hand, the results of the current study were similar to these results, in which there was a moderately good level of awareness among the respondents (69%). When asked if ocular chemical injury can cause ocular complications, the majority of respondents agreed (93.8%), and regarding the immediate action when exposed to ocular chemical injury, the majority (57.8%) said irrigation of the eye with a large amount of water.

Seraj et al. conducted a further study in Saudi Arabia in 2020. This study included 888 participants. All participants were between 18 and 29 years of age. When asked what they would do first if they suffered a chemical eye injury, 697 participants (78.5%) said they would rinse their eyes with water, while 18% would go to the emergency room, 1.2% would use eye drops, and 0.6% would conceal their eyes [17]. Only 8% of respondents disagreed that an alkaline solution should be used to cleanse an eye of an acidic substance. These results are consistent with the present study's findings that the most common age range is between 21 and 30 years (45.1%) and that the majority of respondents who were asked what they would do in the event of ocular chemical harm (53.8%) said they would flush their eyes with water. When asked what they would use to purge an alkaline or acidic chemical from their eyes, the majority of respondents (68.5%) said water.

Lastly, a study was conducted in Ethiopia in 2019 by Asgedom et al. The mean age of the participants was 28 years. The findings indicate that permanent staff members exhibited a higher proportion of responses pertaining to comprehension of 10 out of 12 issues related to chemical dangers, as well as attitudes towards 6 out of 11 of these topics, in comparison to their temporary counterparts. Regarding knowledge ratings, permanent employees demonstrated significantly higher scores (M = 3.7) compared to temporary workers (M = 1.3) (p < 0.001). The present investigation revealed that 69% of participants exhibited a moderately high degree of consciousness. Furthermore, a statistically significant association between age and awareness was identified, with a P-value of 0.000001 [18].

The significant constraint and limitations in this investigation was the employment of observational cross-sectional design which can only establish the relations between factors but not causalities. Secondly, since the questionnaires were issued online, it was difficult to discover insights or observe the behavior of respondents in their natural settings. Also, a larger sample size would have generated more precise results but it wouldn’t be easy to get many respondents within the set period of time. Additionally, the study findings cannot be generalized to the entire Saudi Arabia population considering that it was conducted in only one region.

Recommendations

Expanding the studies’ findings and developing suitable interventions, like health awareness campaigns about ocular chemical damage, should be done in order to prompt corrective measures, social media should have a bigger role in raising awareness and knowledge about chemical ocular injuries and how to act after them, More studies on this topic should be conducted to support this evidence and raise awareness.

## Conclusions

Overall summary, this study’s findings suggest that there exists a moderate degree of knowledge among the public regarding chemical injuries to the eye and the appropriate rapid response to such injuries. Majority of the community members were aware that ocular chemical injury can cause ocular complications, blindness, perforation, scarring and cataracts. Additionally, Chloride and detergents were found to be the most common causes of ocular injuries among the population. The study found the most common immediate action when exposed to ocular chemical injury was to irrigate the eye with large amount of water. The study established a statistically significant association between the age and awareness about ocular chemical injury; thus, age appears to be a key factor influencing the amount of awareness seen. However, it is possible to enhance awareness by the implementation of additional national research within the Saudi region.

## References

[1] Soleimani M, Naderan M (2020). Management strategies of ocular chemical burns: Current perspectives. Clin Ophthalmol.

[2] Singh P, Tyagi M, Kumar Y, Gupta KK, Sharma PD (2013). Ocular chemical injuries and their management. Oman J Ophthalmol.

[3] Xiang H, Stallones L, Chen G, Smith GA (2005). Work-related eye injuries treated in hospital emergency departments in the US. Am J Ind Med.

[4] Merle H, Gérard M, Schrage N (2008). Ocular burns. (Article in French). J Fr Ophtalmol.

[5] Pargament JM, Armenia J, Nerad JA (2015). Physical and chemical injuries to eyes and eyelids. Clin Dermatol.

[6] Bore M (2018). Emergency management: Chemical burns. Community Eye Health J.

[7] (2023). Treating Acute Chemical Injuries of the Cornea. https://www.aao.org/eyenet/article/treating-acute-chemical-injuries-of-cornea.

[8] Desai P, MacEwen CJ, Baines P, Minassian DC (1996). Epidemiology and implications of ocular trauma admitted to hospital in Scotland. J Epidemiol Community Health.

[9] Al-Ghadeer H, Al Amry M, Aldihan KA, Alobaidan OS, AlQahtani GM, Khandekar R (2022). Demographic, clinical profile and management outcomes of ocular chemical injuries in Saudi Children. Clin Ophthalmol.

[10] Sahu S, Karn RR, Ram D, Malla T, Chaudhary S, Singh SK (2021). Comparative study on knowledge and awareness of common ocular diseases among rural and urban community in Siraha district of Nepal: The Lahan Study. Nepal J Ophthalmol.

[11] Dhabaan WA, Almutairi KH, Alzahrani AA (2021). Assessing knowledge and practice about eye injuries first aid, with awareness about the importance of early management among general population in Asser Region, 2020. J Family Med Prim Care.

[12] AlQahtani FA, Alanazi MA, Alanazi MK, Alshalhoub KS, Alfarhood AA, Ahmed SM (2019). Knowledge and practices related to burn first aid among Majmaah community, Saudi Arabia. J Family Med Prim Care.

[13] Alqassim AY, Shami MO, Sabah SA (2022). Community-level awareness of proper immediate steps regarding ocular chemical injury in the Jazan region, Saudi Arabia. Heliyon.

[14] Zhang W, Wood S (2022). Awareness of age-related change, chronological age, subjective age and proactivity: An empirical study in China. Front Psychiatry.

[15] Bamahfouz A, Bakry SM, Alsharif AM, Alomeri S, Alsharif EF, Zamzami OS, Emorsy S (2023). Ocular chemical injuries in Western Saudi Arabia: A study of the public's level of knowledge and experience. Cureus.

[16] Alhothali AS, Aljabri MK, Zamzami OS, Althubaiti MA, Alshanbari AS, Alsaeedi AK, Al-Ghamdi A (2023). Assessing the perceptions and practices toward eye injuries first aid among general population in the Western Region of Saudi Arabia: A cross-sectional study. Cureus.

[17] Seraj H, Khawandanh S, Fatani A, Saeed A, Alotaibi G, Basheikh A (2020). Population-level investigation of the knowledge of ocular chemical injuries and proper immediate action. BMC Res Notes.

[18] Asgedom AA, Bråtveit M, Moen BE (2019). Knowledge, attitude and practice related to chemical hazards and personal protective equipment among particleboard workers in Ethiopia: a cross-sectional study. BMC Public Health.

